# Decision-Making During Percutaneous Coronary Intervention Guided by Optical Coherence Tomography: Insights From the LightLab Initiative

**DOI:** 10.1161/CIRCINTERVENTIONS.122.011851

**Published:** 2022-11-15

**Authors:** Brian Bergmark, Luis A.P. Dallan, Gabriel T.R. Pereira, Julia F. Kuder, Sabina A. Murphy, Jana Buccola, Jason Wollmuth, John Lopez, Joia Spinelli, Jennifer Meinen, Nick E.J. West, Kevin Croce

**Affiliations:** CTO/Complex Coronary Intervention Program, Brigham and Women’s Hospital, Harvard Medical School, Boston, MA (B.B., K.C.).; Thrombolysis in Myocardial Infarction (TIMI) Study Group, Boston (B.B., J.F.K., S.A.M.).; University Hospitals Cleveland Medical Center, OH (L.A.P.D., G.T.R.P.).; Abbott Vascular, Santa Clara, CA (J.B., J.S., J.M., N.E.J.W.).; Providence St. Vincent Hospital, Portland, OR (J.W.).; Loyola University Medical Center, Maywood, IL (J.L.).

**Keywords:** decision-making, intravascular imaging, optical coherence tomography, percutaneous coronary intervention, planning

## Abstract

Use of intracoronary imaging is associated with improved outcomes in patients undergoing percutaneous coronary intervention (PCI). Yet, the impact of intracoronary imaging on real-time physician decision-making during PCI is not fully known.

**METHODS:**

The LightLab Initiative is a multicenter, prospective, observational study designed to characterize the use of a standardized optical coherence tomography (OCT) workflow during PCI. Participating physicians performed pre-PCI and post-PCI OCT in accordance with this workflow and operator assessments of lesion characteristics and treatment plan were recorded for each lesion based on angiography alone and following OCT. Physicians were categorized as having low (n=15), intermediate (n=13), or high (n=14) OCT use in the year preceding participation.

**RESULTS:**

Among 925 patients with 1328 lesions undergoing PCI, the prescribed OCT workflow was followed in 773 (84%) of patients with 836 lesions. Operator lesion assessment and decision-making during PCI changed with OCT use in 86% (721/836) of lesions. Pre-PCI OCT use changed operator decision-making in 80% of lesions, including lesion assessment (45%), vessel preparation strategy (27%), stent diameter (37%), and stent length (36%). Post-PCI OCT changed stent optimization decision-making in 31% of lesions. These findings were consistent across strata of physician prior OCT experience.

**CONCLUSIONS:**

A standardized OCT workflow impacted PCI decision-making in 86% of lesions, with a predominant effect on pre-PCI lesion assessment and planning of treatment strategy. This finding was consistent regardless of operator experience level and provides insight into mechanisms by which intravascular imaging might improve PCI outcomes.

**What is Known**

- Visual estimation of luminal dimensions by angiography during percutaneous coronary intervention (PCI) is often inaccurate and provides little information on plaque morphology, vascular remodeling, or atherosclerosis burden.
- Intracoronary imaging with intravascular ultrasound or optical coherence tomography (OCT) allows for more granular lesion and stent assessment, and intravascular ultrasound-guidance for PCI has shown improved long-term outcomes in randomized trials. Nonetheless, adoption of intracoronary imaging during PCI remains low in the United States, and the impact of OCT on intraprocedural decision-making in contemporary practice is incompletely understood.

**What the Study Adds**

- The LightLab Initiative aims to document real-world use of OCT by collecting data on changes in procedural decision-making during PCI using a standardized OCT workflow.
- Compared with angiography alone, OCT changed procedural decision-making in 86% of lesions, including in 80% of pre-PCI OCT pullbacks. In nearly one-third of lesions, post-PCI OCT identified an issue requiring further optimization.
- These findings were consistent regardless of an operator’s prior experience with OCT.

**See Editorial by Razzouk and Attubato**

The limitations of angiography for coronary lesion assessment are well established.^1,2^ Visual estimation of luminal dimensions is often inaccurate and angiography provides little to no information on plaque morphology, vascular remodeling, or atherosclerosis burden.^3–7^ Angiography is also insensitive for detection of suboptimal stent results, including underexpansion, malapposition, and edge dissection.^1,8–11^ Intracoronary imaging with intravascular ultrasound or optical coherence tomography (OCT) allows for more granular lesion and stent assessment, and intravascular ultrasound-guidance for PCI has shown improved long-term outcomes in randomized trials.^12,13^

Nonetheless, adoption of intracoronary imaging during PCI is <10% in the United States, and the impact of these modalities on intraprocedural decision-making in contemporary practice is incompletely understood.^14–16^ The LightLab Initiative was developed to assess the procedural impact of a prescriptive OCT workflow during PCI in a real-world, unselected patient population.

## Methods

### Data Sharing

Because of the sensitive nature of the data collected for this study, requests to access the dataset from qualified researchers trained in human subject confidentiality protocols may be sent to Dr Brian Bergmark, Thrombolysis in Myocardial Infarction (TIMI) Study Group, Brigham and Women’s Hospital, Boston, MA.

### Study Design Overview

**Figure 1.**
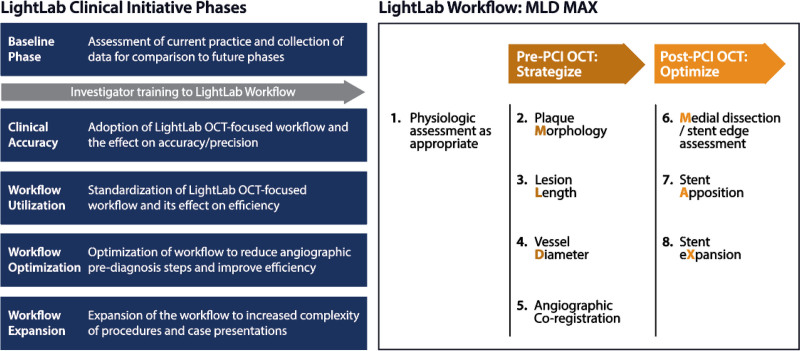
LightLab study phases and MLD MAX workflow. MLD MAX is a prescriptive optical coherence tomography (OCT) workflow that guides treatment decisions during percutaneous coronary intervention (PCI).^17^ The workflow encompasses assessment of lesion morphology, length, and diameter from the pre-PCI OCT pullback, and medial dissection, stent apposition, and stent expansion from the post-PCI OCT pullback (acronym MLD MAX).

The LightLab Initiative (“LightLab”) is a multicenter, prospective, observational study performed in multiple phases and designed to characterize the influence of a standardized OCT workflow on physician decision-making and procedural efficiency (Figure 1). The workflow facilitates the prescriptive utilization of OCT data to guide treatment decisions during PCI, encompassing assessment of lesion Morphology, Length, and Diameter from the pre-PCI OCT pullback, and Medial dissection, stent Apposition, and stent eXpansion from the post-PCI OCT pullback (acronym MLD MAX; Figure 1). MLD MAX was developed by a consortium of academic cardiologists and has been described previously.^17^ Thresholds for change in procedural decision-making, such as alteration in vessel preparation strategy in response to OCT identified calcium, were at the discretion of the physician and were not prespecified. The LightLab decision-making phase presented here was conducted at 16 US hospitals with 42 participating physicians and has received Institutional Review Board approval or exemption at all centers. Participating physicians were trained on the MLD MAX workflow by an Abbott OCT Field Clinical Engineer (FCE) regardless of prior OCT experience. The LightLab Initiative was designed through an industry/academic collaboration and the study was sponsored by Abbott. LightLab was managed by Abbott and the study database was separately and independently housed at Abbott and within the Thrombolysis in Myocardial Infarction (TIMI) Study Group at the Brigham and Women’s Hospital. All analyses in this article were performed by the TIMI Study Group on an independently held dataset. Co-authors contributed to the design of the study, data collection, and/or design of planned analyses. The current article was drafted and submitted by BB and KC. The LightLab Investigators are listed in the Supplemental Material.

### Data Collection and Procedures

Procedural data and independent physician angiogram, lesion, and OCT assessments were collected and entered in real time by FCEs onto a tablet computer using a custom data form (Vablet, Tustin, CA) and archived thereafter using Microsoft Azure (Microsoft Corp, Redmond, WA); all data attributes were compliant with HIPAA requirements. Data were collected in all coronary procedures by participating physicians. However, only PCIs with pre- and post-PCI OCT were included in this analysis. All PCIs by participating physicians were eligible, with inclusion contingent on physician discretion as to whether the PCI was clinically appropriate for OCT and the LightLab workflow.

After performing coronary angiography, physicians provided their assessments of lesion type and morphology, lesion length and diameter, and planned treatment strategies (vessel preparation and intended stent diameter/length) to the FCE. Physicians repeated this assessment after performing the pre-PCI OCT pullback. Stent diameter was sized using external elastic lamina measurements when available, and stent length was selected to provide full lesion coverage while avoiding landing zones in high-risk morphologies. Differences between the angiography-based and OCT-based assessments were considered a change. Following treatment of the lesion, the physician performed a second, post-PCI OCT pullback. A change here was defined as requirement for further vessel optimization (additional postdilatation or stenting). The recommended threshold for adequate stent expansion was minimum expansion ≥90% or minimum stent area ≥4.5 mm^2^. If further optimization was performed after the post-PCI OCT, subsequent OCT after optimization was encouraged, but not required. In the case that more than one OCT pullback was performed after PCI, the final OCT was used as the post-PCI OCT. Table S1 provides an overview of the data collected to compare OCT guidance with angiographic guidance.

Baseline data for each operator were collected prior to the introduction of the MLD MAX workflow, and physician experience with OCT in the year preceding participation was classified as low (<15 OCT procedures; n=15), intermediate (15 to <50 OCT procedures; n=13), or high (*≥*50 OCT procedures; n=14). Physicians used commercially approved products during PCI procedures at their discretion and in accordance with their standard of care. The LightLab workflow uses OCT (ILUMIEN OPTIS, OPTIS Integrated, and OPTIS Mobile systems with Dragonfly OPTIS Imaging Catheters) and physiology (PressureWire X Guidewire, compatible with the OPTIS Imaging or QUANTIEN Measurement systems) within the approved indications for use.

### Statistical Analysis

Proportions are presented as n/N and % with comparisons made between groups using the χ^2^ test. Continuous variables were compared using the Wilcoxon rank sum test. All analyses were performed by the TIMI Study Group on an independently held dataset. Analyses were performed using SAS Version 9.4 (SAS Institute, Cary, NC), and a *P*<0.05 was considered significant for all comparisons with no adjustment for multiple testing.

## Results

**Figure 2.**
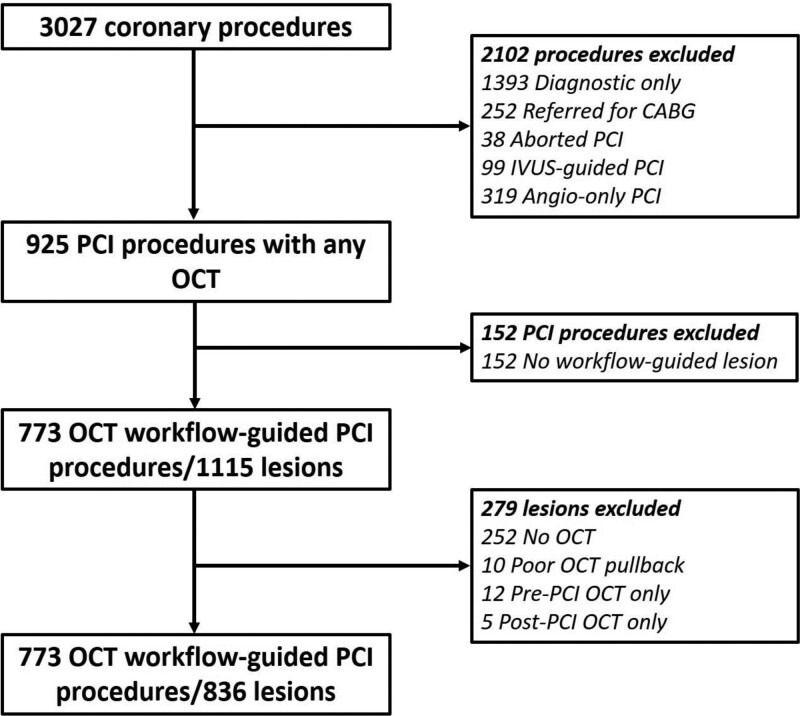
Study flowchart: LightLab Initiative decision-making phase. CABG indicates coronary artery bypass grafting; IVUS, intravascular ultrasound; OCT, optical coherence tomography; and PCI, percutaneous coronary intervention.

Between January 2019 and March 2020, data were gathered from 3027 procedures. Of 925 OCT procedures (1328 lesions), a total of 773 of these procedures were LightLab-qualifying PCIs, with 836 lesions treated according to the workflow (Figure 2). Procedure and lesion characteristics are presented in Table 1 and are further stratified by low, intermediate, and high OCT operator experience as summarized in Table 2. The majority of procedures were performed via radial access (62%), in single vessel/single lesion disease (76%), and with an urgent/non-elective indication (70%). Half (50%) of the cases were performed in the left anterior descending coronary artery, 29% in the right coronary artery, and 15% in the left circumflex. Physiological assessment was performed in 13% of the lesions. Operator-reported lesion complexity was high, reflected in the prevalence of American College of Cardiology/American Heart Association type C (57%) and long (*≥*28 mm) lesions (46%). In-stent restenosis (17%), bifurcation disease (9%), and chronic total occlusions (3%) were present in a minority of lesions.

**Table 1.**
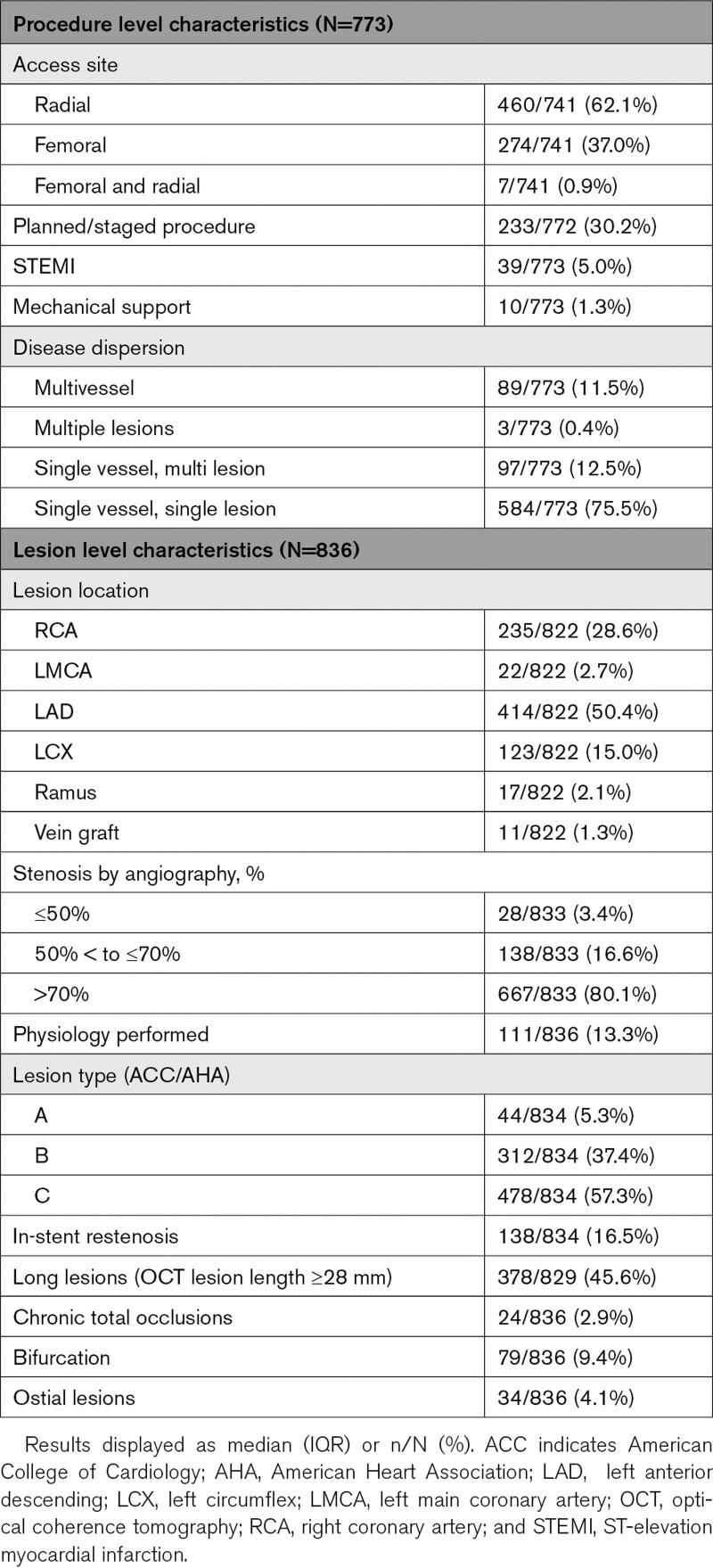
Procedure and Lesion Characteristics for the Full Study Cohort

**Table 2.**
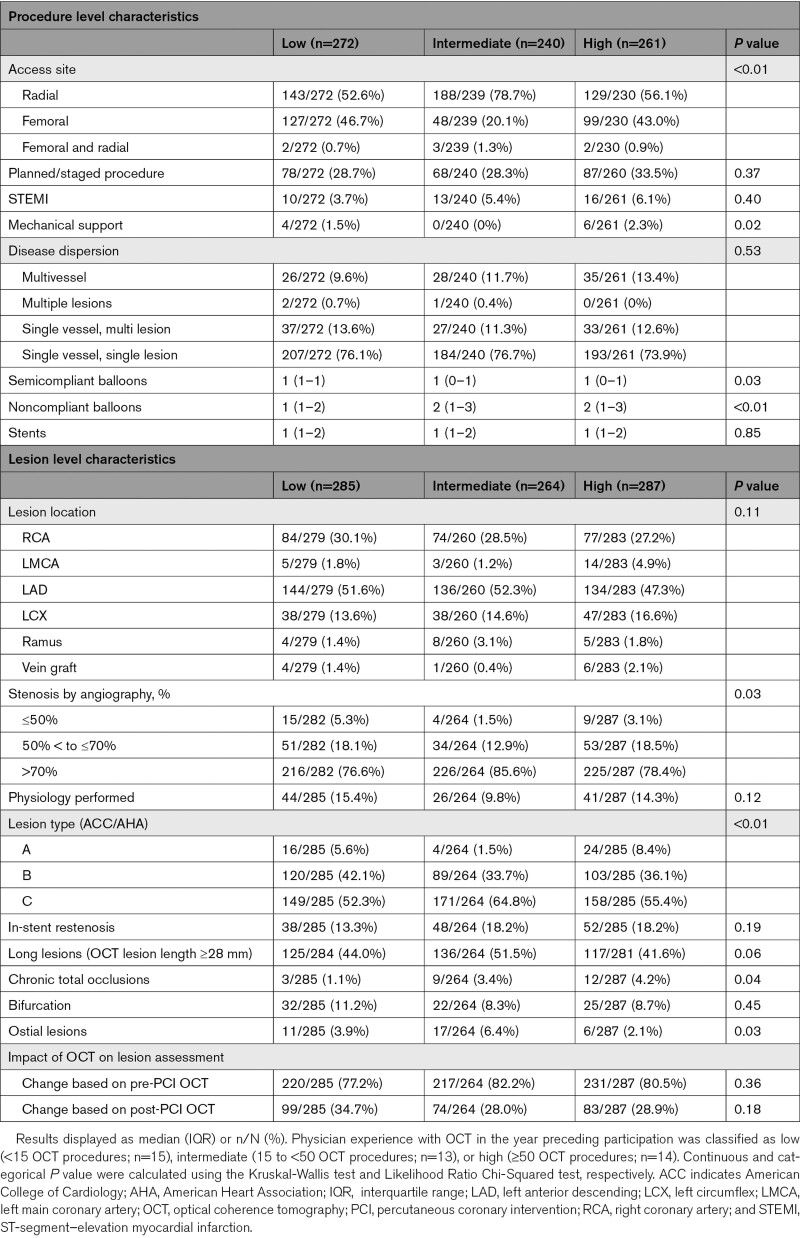
Procedure and Lesion Characteristics and Impact of OCT on Decision-Making by Operator Experience

### Impact of OCT on Procedural Decision-Making

**Figure 3.**
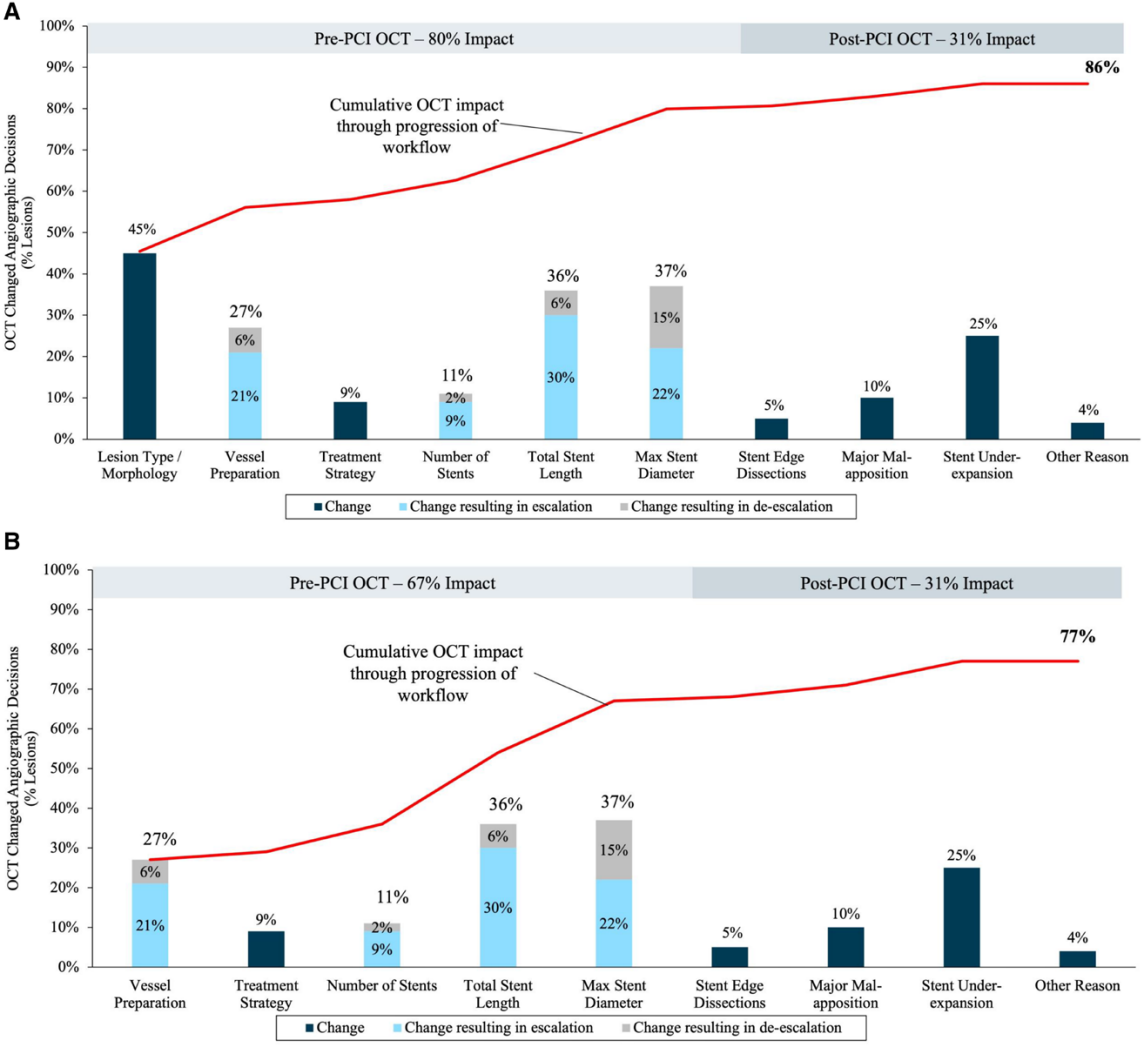
Impact of optical coherence tomography (OCT) workflow guidance on changes in lesion assessment and percutaneous coronary intervention (PCI) decision-making. **A**, Bar chart indicates % of lesions studied where OCT altered initial physician decision-making based on angiographic assessment; bars are colored according to a definitive change in assessment (dark blue bars: ie, lesion morphology, treatment strategy and presence of stent edge dissections, major malapposition or stent underexpansion), or where strategy was escalated (mid blue bars: ie, more aggressive vessel preparation, increase in stent number/length/diameter) or de-escalated (gray bars: that is, less aggressive/no vessel preparation, reduction in stent number/length/diameter). The % change in strategy is indicated by the accompanying legend. Cumulative impact of changes in decision-making per lesion are demonstrated by the red trend line and indicate an overall impact of OCT on changes from angiographic decision-making in 86% of the cases. The bars are grouped by pre-PCI impact (cumulative 80% lesions) and post-PCI impact (cumulative 31% lesions). Note: additional factors were assessed within the diagnostic category and not shown on graph: lesion type per American College of Cardiology/American Heart Association guidelines (21%) and decision to treat percutaneously (2%). **B**, Focused impact of OCT on procedural decision-making excluding effects on lesion assessment.

The MLD MAX OCT workflow changed lesion assessment and procedural decision-making in 86% (721/836) of lesions (Figure 3A; Table 3). There was a change in assessment based on the pre-PCI OCT in 80% (n=668) of lesions and a change based on the post-PCI OCT in 31% (n=256). After removing OCT’s impact on lesion type/morphology assessment, the MLD MAX OCT workflow changed procedural decision-making in 77% (647/836) of lesions (Figure 3B).

**Table 3.**
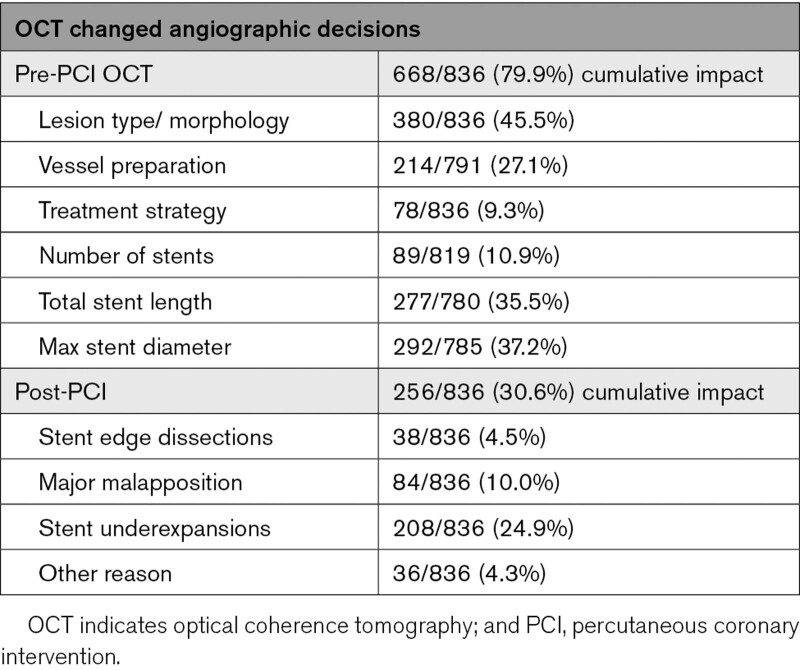
Impact of OCT on Lesion Assessment and PCI Procedural Decision-Making

**Figure 4.**
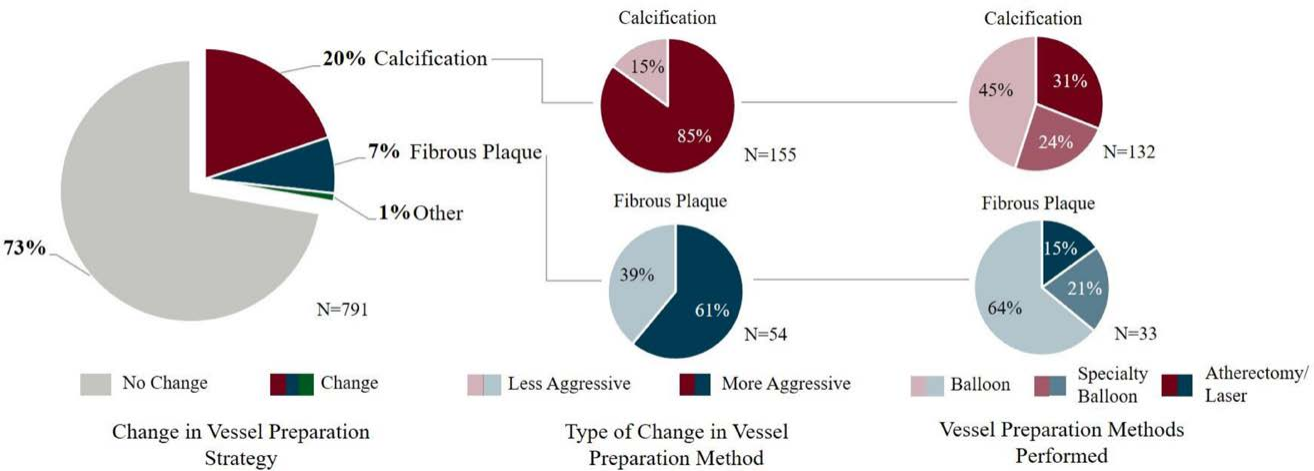
Impact of optical coherence tomography (OCT) guidance on changes in vessel preparation modality categorized by predominant lesion morphology. Data indicate frequency of change in vessel preparation strategy categorized by predominant lesion morphology assessed by OCT. Vessel calcification identified on OCT drove most changes in strategy, with increased use of specialty balloons or atherectomy.

Regarding the pre-PCI OCT, changes in operator assessment of lesion morphology (n=380; 45%) were frequent. Pre-PCI OCT led to a change in vessel preparation strategy in 27% of lesions, with an escalation in device selection seen in the majority of these instances (21% of lesions) and de-escalation based on the OCT seen in 6% of lesions. When a change in vessel preparation strategy was selected, calcification was the predominant morphology identified (20%, 155/791) compared with fibrous plaque (7%, 54/791; Figure 4). Severity of lesion calcification was often angiographically underestimated, as 85% (132/155) of the calcified lesions underwent a more aggressive form of vessel preparation than originally planned based on the angiogram alone. Fifty-five percent were treated using atherectomy, laser, or specialty balloon, whereas the other 45% were upgraded to a noncompliant balloon from a semi-compliant balloon, or to a balloon from no initial plan for vessel preparation.

In most cases (89%, 730/819), the number of stents planned for implantation was unchanged; when the number of planned stents was increased after OCT guidance (9%, 74/819), it was primarily due to changes to planned stented length. Overall planned stent length changed by more than ±5 mm in 36% (277/780) of lesions, with a planned length increase in 30% (232/780) of lesions. Planned stent diameter changed by more than ±0.25 mm in 37% (292/785) of lesions, with more common under- (23%) rather than oversizing (15%) with angiography alone.

Overall, the LightLab workflow achieved an average minimum stent expansion of 80±14.0% in lesions where the prescribed workflow was utilized (N=833 with available minimum stent expansion values). In line with the prescriptive MLD MAX workflow, 82% (685/833) of lesions underwent post-dilatation prior to the post-PCI OCT pullback, with an average minimum stent expansion of 80±13.7% in lesions with pre-OCT post-dilatation. Physicians performed further targeted optimization in a subset of lesions (52%, 430/833) after the post-PCI OCT assessment, with the post-PCI OCT run identifying underexpansion in 25% of lesions (208/836), major malapposition in 10% (84/836), and edge dissection in 5% (38/836). Among lesions that underwent further optimization, average minimum expansion was 76±14.1% prior to optimization. In lesions where additional optimization was not performed (48%, 403/833), the average minimum expansion was 84±12.7%.

### Prior Physician Experience

**Figure 5.**
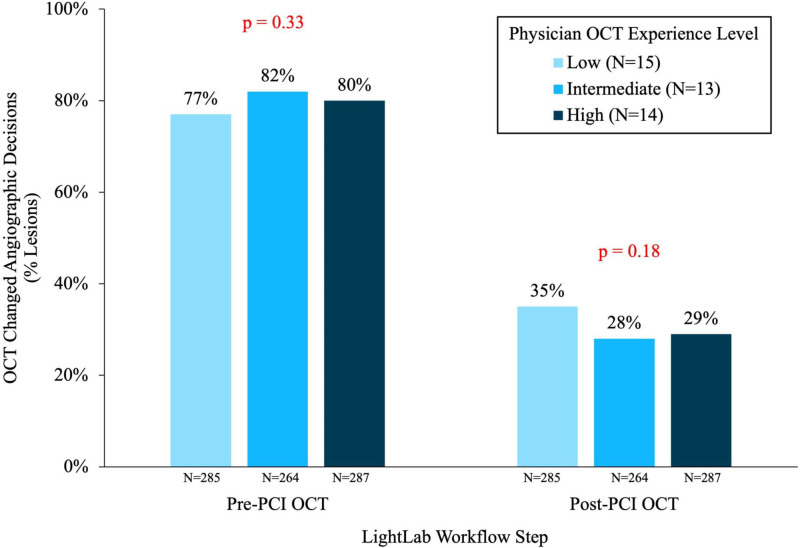
Prior physician optical coherence tomography (OCT) experience and changes in decision-making for pre- and post-percutaneous coronary intervention (PCI) OCT imaging. Bar chart demonstrates frequency of change to angiographic decision-making/lesion assessment after pre-PCI and post-PCI imaging with OCT, and demonstrates the lack of impact of self-reported prior physician OCT experience (see Figure 2) in reducing utility of OCT imaging.

The median number of OCT procedures performed in the year preceding LightLab participation was 6 (IQR 3–7) for the low experience physicians, 25 (IQR, 15–26) for the intermediate experience physicians, and 84 (50–110) for the most experienced physicians. Procedure and lesion characteristics were overall similar when categorized according to degree of operator prior OCT experience (Table 2). The impact of OCT on procedural decision-making was consistent regardless of prior OCT experience, with an impact of pre-PCI OCT in 77%, 82%, and 80% of lesions (*P*=0.33) and an impact of post-PCI OCT in 35%, 28%, and 29% of lesions (*P*=0.18) for low, intermediate, and high experience operators, respectively (Table 2; Figure 5).

## Discussion

These analyses from the multicenter LightLab collaboration have yielded several principal findings: (1) OCT guidance impacted lesion assessment and physician decision-making in 86% of PCI’s in this real-world all-comers population; (2) the greatest difference in operator assessment between angiography and OCT-based decision-making was with the pre-PCI OCT; (3) in nearly one-third of lesions, post-PCI OCT identified an issue requiring further optimization; and (4) these findings were consistent regardless of an operator’s prior experience with OCT.

The granularity and volume of this dataset are distinct and demonstrate a large impact of OCT on lesion assessment, procedural planning, and stent optimization when compared with angiography alone. These findings highlight the importance of procedure planning by indicating that most of the assessments made during a PCI, such as lesion length and need for adjunctive treatments before stenting, are measurably impacted by OCT.

In almost one-third of cases, physicians changed vessel preparation strategy according to the vessel and lesion anatomy. In this cohort, severity of lesion calcification was often angiographically underestimated, and when accurately quantified resulted in changes to a more aggressive vessel preparation device and/or strategy in 85% of calcified lesions. Of these lesions, 45% resulted in pre-dilatation with semi- or noncompliant balloons, 24% resulted in cutting or scoring balloons, and 31% resulted in rotational/orbital or laser atherectomy. The importance of the often-neglected pre-PCI OCT pullback^18,19^ is highlighted by these data and emphasizes the role of complete vessel evaluation and lesion preparation for procedural success, particularly to achieve optimal stent expansion.

Additionally, these data show that the pre-PCI OCT pullback is crucial to accurately determine appropriate stent sizes, as angiographic assessment led to suboptimal stent size planning (diameter and length) in over one-third of lesions. These findings are consistent with previous studies^1,14,20^ and underscore that intravascular imaging is more accurate than angiography. Previous data have demonstrated that intracoronary imaging–guided predilation, stent sizing, and postdilation are associated with a lower risk of cardiac events, including in complex lesions,^10,18,19^ which is particularly relevant given the now-common use of PCI to treat complex lesions and stent failure.^21^ Further, after removing lesion type/morphology assessment from the calculation of cumulative OCT impact, OCT still changed procedural decision making in greater than three quarters of lesions.

When considering post-stent assessment, the population of lesions that followed the LightLab workflow achieved on average 80% minimum stent expansion before further optimization was performed. First, this finding demonstrates favorable procedural outcomes with a standardized imaging-based workflow. Second, OCT is able to identify issues associated with future stent failure, including underexpansion, major malapposition and edge dissection that angiography alone may miss. Stent underexpansion is a powerful predictor of early stent thrombosis and restenosis after DES implantation,^10,22–27^ and observational data show a correlation between ≥80% stent expansion and lower rates of major adverse cardiac events.^28^ Whether the standardized OCT workflow evaluated here truly leads to improved long-term clinical outcomes is being investigated in the ongoing ILUMIEN-IV OPTIMAL PCI randomized trial (NCT03507777).

Finally, the consistent findings across the spectrum of physician experience are relevant. There is often a perception that frequent intracoronary imaging users become “trained” and develop lesser reliance on the imaging data over time. The data shown here do not support this notion; instead, these findings suggest an intrinsic and persistent limitation in angiography-based vessel and lesion assessment.

### Study Limitations

These data should be interpreted with several caveats. First, the study lesions were selected by physicians as suitable for OCT workflow which may introduce bias and limit generalizability. Second, patient clinical variables and demographic data were not collected, which may limit the understanding of population characteristics and their relevance to other groups. Finally, the presence of an FCE collecting procedural data may have influenced OCT interpretation and operator behavior, and concurrent data entry during procedures may be more prone to error than in other settings.

### Conclusions

In conclusion, a standardized OCT workflow impacted lesion assessment and PCI decision-making in 86% of lesions, with a predominant effect on pre-PCI lesion assessment and planning of treatment strategy. These findings were consistent regardless of operator experience and may provide insight into the association between intracoronary imaging use during PCI and favorable long-term outcomes.

## ARTICLE INFORMATION

### Acknowledgments

We would like to acknowledge all participating LightLab Physician Champions for their contributions to the data presented, and the LightLab Field Clinical team, instrumental to data collection for this publication.

### Sources of Funding

The LightLab Initiative is a clinical program funded by Abbott Vascular.

### Disclosures

Dr Bergmark received research grants (through the Brigham and Women’s Hospital) from Pfizer, Ionis, Quark, AstraZeneca/MedImmune, Amgen; Consulting/personal fees: Philips, Abbott Vascular, CSI, Abiomed, Servier, Janssen, Quark, Daiichi Sankyo. J. Buccola was employed by Abbott Vascular. Dr Wollmuth received honoraria from Abbott Vascular, Boston Scientific, Abiomed, Cardiovascular Systems, Inc, Philips, and Asahi Intecc. J. Spinelli was employed by Abbott Vascular. J. Meinen was employed by Abbott Vascular. Dr West was employed by Abbott Vascular. Dr Croce received grant support from Abbott, Takeda, Teleflex, CSI, honoraria from Abbott, Biotronik, Philips, Abiomed, CSI, Takeda and Cordis and is a major stock shareholder in Dyad Medical. Dr Bergmark, J.F. Kuder, and S.A. Murphy are members of the TIMI Study Group, which has received grant support from: Abbott, Amgen, Anthos Therapeutics, AstraZeneca, Bayer HealthCare Pharmaceuticals, Inc, Daiichi-Sankyo, Eisai, Intarcia, MedImmune, Merck, Novartis, Pfizer, Quark Pharmaceuticals, Regeneron Pharmaceuticals, Inc, Roche, Siemens Healthcare Diagnostics, Inc, The Medicines Company, Zora Biosciences.

### Supplemental Material

Table S1

LightLab Investigators

## Supplementary Material


