# Supplementary material for: Decision-Making During Percutaneous Coronary Intervention Guided by Optical Coherence Tomography: Insights From the LightLab Initiative
Source: Circ Cardiovasc Interv. 2022 Nov 15;15(11):872–81. doi: 10.1161/CIRCINTERVENTIONS.122.011851 (PMC9648988; doi:10.1161/CIRCINTERVENTIONS.122.011851)
Supplement: Supplementary file 1 [file hcv-15-872-s001.pdf]

Supplemental Material

Decision-Making During Percutaneous Coronary Intervention Guided by Optical Coherence Tomography: Insights from the LightLab Initiative

Supplemental Table S1. Data collected to inform changes in physician decision-making in the LightLab Initiative. OCT pullback (pre-/post-PCI), procedure steps assessed, nature of assessment noted with drop-down menu options available to FCE during PCI procedures, and criteria used to identify a change in decision-making for analysis.

Pullback	Procedure Step	Assessment	Available Options			Criteria for Change
Pre-PCI	Diagnosis	Lesion Type	• Type A	• Type B	• Type C	Any difference
		Lesion Evaluation<br><br>(inclusive of lesion type choices and ISR type changes between Angio and OCT)	• Discrete (<10 mm length)<br>• Concentric<br>• Readily accessible<br>• Non-angulated segment <45°<br>• Smooth contour<br>• Little or no calcification<br>• Less than total occlusive<br>• Not ostial in location<br>• No major branch involvement<br>• Absence of thrombus	• Tubular (10 – 20 mm length)<br>• Eccentric<br>• Moderate tortuosity of proximal segment<br>• Moderately angulated segment, 45° - 90°<br>• Irregular contour<br>• Moderate to heavy calcification<br>• Ostial in location<br>• Bifurcation lesions requiring double guidewires<br>• Some thrombus present<br>• Total occlusion < 3 months old	• Type C Lesions<br>• Diffuse (>2 cm length)<br>• Excessive tortuosity of proximal segment<br>• Extremely angulated segments, >90°<br>• Inability to protect major side branches<br>• Degenerated vein graft with friable lesions<br>• Total occlusion >3 months old<br>• Severe calcification	Any difference

Pullback	Procedure Step	Assessment	Available Options			Criteria for Change
			ISR Type	• NIH – fibrotic/lipidic<br>• Edge-stent restenosis	• Under-expansion<br>• NIH – calcific	
		Treat Percutaneously	• Yes	• No	• Undecided	Any difference
	Treatment	Vessel Prep	• Compliant Balloon<br>• Non-Compliant Balloon<br>• Cutting Balloon	• Scoring Balloon<br>• Orbital<br>• Rotational<br>• Thrombus Aspiration	• Laser<br>• Shockwave/ Coronary Lithotripsy	Any difference (Note: vessel prep performed before pre-PCI OCT was not considered a change.)
		Treatment	• Direct Stent<br>• Stent	• POBA<br>• Other Treatment		Any difference
		Number of Stents	Record value			Any difference
		Max Stent Diameter	Record value	Note: LightLab workflow suggested sizing per ILUMIEN IV protocol, use EEL (if visible) and size down to the nearest 0.25mm stent, or lumen (if EEL not visible) and size up to the nearest 0.25mm stent		More than ±0.25 mm of max value recorded for all stents
		Total Stent Length	Record value			More than ±5 mm for total stent length
Post-PCI	Optimization	Malapposition	• None	• Minor	• Major	Major & reaction
		Under Expansion	Record value			≤80% & reaction
		Edge Dissection	Record presence of dissection			Presence & reaction
		Geographic Miss	Record untreated reference segment disease			Presence & reaction
		Other	Record intra-stent thrombus/plaque protrusion			Presence & reaction

OCT=optical coherence tomography; PCI=percutaneous coronary intervention; FCE=field clinical engineer; ISR=in-stent restenosis; NIH=neointimal hyperplasia; EEL=external elastic lamina; POBA= ‘plain old’ balloon angioplasty.

LightLab Investigators:

Dr. Ethan Korngold
Dr. Jason Wolmuth
Dr. Richard Sohn
Dr. Bimal Padaliya
Dr. David Rizik
Dr. Kethes Waram
Dr. Thomas McMinn
Dr. Frank Zidar
Dr. Bassem Chehab
Dr. Aziz Maksoud
Dr. Matheen Khuddus
Dr. Mark Tulli
Dr. Chris Caputo
Dr. Hiram Bezerra
Dr. William Wolf
Dr. Michael Johnson
Dr. Judah Rauch
Dr. Kevin Croce
Dr. Brian Bergmark
Dr. Steven Roark
Dr. Jay Koons
Dr. Mark Greenberg
Dr. Arthur Smith
Dr. Charles Cannan
Dr. Eric Osborn
Dr. Eric Secemsky
Dr. Marie France Poulin
Dr. Hector Tamez
Dr. Zach George
Dr. Josh Doll
Dr. Jesse Jorgensen
Dr. Daniel Spoon
Dr. James “Tod” Maddux
Dr. Mike Reed
Dr. John Lopez
Dr. Amir Darki
Dr. Joseph Henderson
Dr. Chetan Patel
Dr. Mayur Bhakta

Dr. Lowell Steen
Dr. Ajar Kocher
Dr. Dominic Johnson
